# Multimodal X-ray probe station at 9C beamline of Pohang Light Source-II

**DOI:** 10.1107/S1600577522006397

**Published:** 2022-06-27

**Authors:** Daseul Ham, Su Yong Lee, Sukjune Choi, Ho Jun Oh, Do Young Noh, Hyon Chol Kang

**Affiliations:** a Department of Material Science and Engineering, Chosun University, Gwangju 61452, Korea; b Pohang Accelerator Laboratory, POSTECH, Pohang 37673, Korea; c Department of Physics and Photon Science, Gwangju Institute of Science and Technology, Gwangju 61005, Korea

**Keywords:** multimodal X-ray probe, *in situ* X-ray diffraction, four-point probe station, electrical property, beamline

## Abstract

In this study, the conceptual design and performance of a multimodal X-ray probe station recently installed at the 9C coherent X-ray scattering beamline of the Pohang Light Source-II are presented. The purpose of this apparatus is to measure coherent X-ray diffraction, X-ray fluorescence and electrical properties simultaneously. A miniature vacuum probe station equipped with a four-point probe was mounted on a six-axis motion hexapod. This can be used to study the structural and chemical evolution of thin films or nanostructures, as well as device performance including electronic transport properties. This probe station also provides the capability of varying sample environments such as gas atmosphere using a mass-flow-control system and sample temperatures up to 600°C using a pyrolytic boron nitride heater. The *in situ* annealing of ZnO thin films and the performance of ZnO nanostructure-based X-ray photodetectors are discussed. These results demonstrate that a multimodal X-ray probe station can be used for performing *in situ* and *operando* experiments to investigate structural phase transitions involving electrical resistivity switching.

## Abstract 2

A multimodal X-ray probe station has been developed and commissioned at the 9C coherent X-ray scattering beamline of the Pohang Light Source-II. Results from the *in situ* annealing of ZnO thin films and the performance of ZnO nanostructure-based X-ray photodetectors are presented for demonstration.

## 1. Introduction

Since the discovery of the nature of X-ray interaction with atoms (Bragg, 1913 ▸; Friedrich *et al.*, 1912 ▸), X-ray-based analytical techniques such as imaging, diffraction/scattering and spectroscopy have been extensively developed and used for probing complex material systems (Holler *et al.*, 2017 ▸; Ice *et al.*, 2011 ▸; Sanchez-Cano *et al.*, 2021 ▸; Timoshenko & Cuenya, 2021 ▸). Over the past decade, multimodal X-ray probes have been utilized in synchrotron facilities to comprehensively understand material properties such as nanoscale atomic structures, magnetic domains and element-specific chemical heterogeneities (Pattammattel *et al.*, 2020 ▸; Martínez-Criado *et al.*, 2014 ▸). With the advent of efficient detectors, scanners and X-ray focusing optical devices at the nanometre scale (Masuda *et al.*, 2017 ▸; Nazaretski *et al.*, 2017 ▸; Kang *et al.*, 2008 ▸), it has become possible to design and integrate a new type of experimental setup known as an all-in-one probe, in which several X-ray-based characterization tools are implemented on a tabletop platform. The near- and far-field scattering profiles and images can be recorded simultaneously using this probe. In addition, this has application in the investigation of the chemical information and electronic structure of materials by measuring X-ray absorption/emission spectra with the tunable energy of synchrotron X-rays and photoelectron spectroscopy simultaneously (Sanchez-Cano *et al.*, 2021 ▸; Timoshenko & Cuenya, 2021 ▸; Kersell *et al.*, 2021 ▸).

Even though multimodal X-ray probes have been efficient, they have certain issues such as the analysis of vast amounts of data, complexity of experiments, and integration of all results from individual techniques (Chen-Wiegart *et al.*, 2020 ▸). Despite these challenges, notable achievements have recently been reported. Multimodal X-ray microscopy combined with electron microscopy has become a powerful analytical tool that complements each technique (Lo *et al.*, 2019 ▸). Furthermore, an early multimodal X-ray probe was upgraded to an *in situ operando* probe, which allows real-time measurements during device operation or the reaction and phase transformation of materials (Bak *et al.*, 2018 ▸). For example, by combining multimodal X-ray techniques, *operando* studies have been performed to determine the structural and chemical evolution of functional additives during the cyclic lithiation and delithiation of Li-ion batteries (Nelson *et al.*, 2012 ▸; Sun *et al.*, 2017 ▸; Singer *et al.*, 2014 ▸). Chen *et al.* (2017 ▸) reported proof of the role of perovskite layers in improving the performance of lead halide perovskite solar cells using *in situ* X-ray diffraction (XRD) combined with electrical device performance measurements. Such studies strongly support the conclusion that multimodal X-ray probes can provide comprehensive conclusions by measuring device performance simultaneously during realistic device operation.

In this study, we combined an X-ray diffractometer with a four-point probe station to assemble a multimodal X-ray probe station at the 9C beamline of Pohang Light Source-II (PLS-II). This can be used to study the structural, element-specific and electrical properties of thin films or nanowires. To demonstrate the performance of the multimodal X-ray probe station, we analyzed the *in situ* annealing of ZnO thin films and the device performance of a ZnO nanostructure-based X-ray photodetector.

## 2. Conceptual design of multimodal X-ray probe station

The 9C beamline used in this study is dedicated to coherent X-ray diffraction imaging (CDI) and microbeam diffraction techniques (Yu *et al.*, 2014 ▸). X-rays are generated from the in-vacuum undulator of the beamline. X-rays are then monochromated using a Si(111) double-crystal monochromator (DCM) located at 17.4 m. A flat mirror for harmonic rejection is installed at 20 m, just behind the DCM. The monochromatic X-rays are finally focused using a Kirkpatrick-Baez (K-B) mirror system. A six-circle X-ray diffractometer is positioned at the focal point of the K-B mirror, which is approximately 30 m from the source. The Eulerian cradle of the diffractometer was modified with a hexapod positioning system to facilitate installing a large sample chamber such as an X-ray probe station.

**Figure 1:**
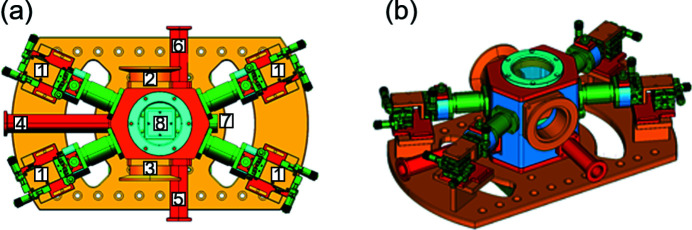
Conceptual design of the miniature X-ray probe station. (*a*) Top view and (*b*) perspective view. It mainly consists of a miniature vacuum chamber and motion stages for the probe holder.

The conceptual design of the miniature vacuum X-ray probe station is illustrated in Fig. 1 ▸. Because the entire probe station could be mounted on top of the precision six-axis hexapod positioning system (PI, H-840), the chamber dimensions were minimized to approximately 8 kg to fulfill the condition of load capacity (approximately 30 kg) of the hexapod manipulator. The probe station mainly consists of motion stages (1) for the probe holder and miniature vacuum chamber with X-ray windows (2, 3), pumping (4), gas inlet (5) and bent ports (6). A vacuum feedthrough port (7) was used to connect electrical power cables for pyrolytic boron nitride (PBN) heaters and K-type thermocouples. A window (8), which is utilized for the X-ray fluorescence detector and vision camera, is located in the upper cap of the chamber.

The probe holders are positioned along the diagonal direction of the hexagonal pillar-shaped chamber and are connected by welded bellows to the external *x*-*y*-*z* stages. The travel length of the linear motion stage was approximately 25 mm in the *x*-, *y*- and *z*-directions. In particular, the rotating center of the diffractometer was positioned 12 mm both horizontally and vertically offset from the center of the X-ray window. Thus, the incident angle through the X-ray windows can be varied by up to approximately 23° in both the horizontal and vertical directions, which corresponds to a maximum X-ray momentum transfer *Q* of 3.19 and 3.87 Å^−1^ at *E* = 8 and 10 keV, respectively.

**Figure 2:**
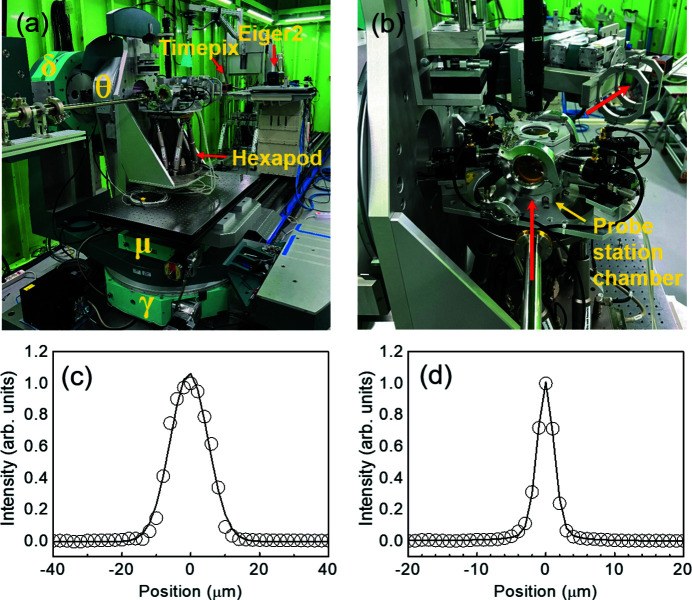
(*a*, *b*) Photographs of the multimodal X-ray probe station that includes the miniature probe station, hexapod manipulator and six-circle X-ray diffractometer. The X-ray diffractometer operates in ‘2 + 2’ mode to access *Q* space by combining motions of two detector circles and two sample circles. Panels (*c*) and (*d*) show intensity profiles at the focal point for estimating the focus size in the horizontal (*c*) and vertical (*d*) directions, respectively.

A hexapod manipulator equipped with an X-ray probe station was then mounted on the bench of the vertical θ circle of a six-circle X-ray diffractometer (Huber 5021), as shown in Figs. 2 ▸(*a*) and 2(*b*). In particular, the ϕ and χ circles of the six-circle diffractometer were replayed using a six-axis hexapod manipulator. The hexapod manipulator also adjusts the sample position for nanometre resolution, which is useful for the alignment of patterned nanoscale thin films or nanowires. The actuator resolution was approximately 16 nm.

Because the brightness of the incident X-rays generated from the undulator was optimized in the range 5–8 keV, in this study an X-ray energy of 8 keV was selected to access the high *Q* range. Incident X-rays in front of a miniature X-ray probe station can be focused using a K-B mirror system located 3 m upstream of the sample. Length, incidence angle and focal length of the K-B mirror are 150 mm, 3 mrad and 3 m, respectively. A K-B mirror is optimized to have a divergence of 0.15 mrad. Ideally, it is designed to focus X-rays smaller than 1 µm at the sample position in both the horizontal and vertical directions. However, instrumentation errors, such as source demagnification, coherent volume, monochromator crystal and vibration, of the source and beamline components limit the actual beam size to 13 µm (h) × 3 µm (v) at the sample position estimated from the intensity profiles shown in Figs. 2 ▸(*c*) and 2(*d*), respectively. In particular, emittance of the X-ray source of the PLS-II is too large to achieve the designed focus, especially in the horizontal direction. Submicrometre focus can be achieved by defining a virtual source using a slit system located in front of the DCM, but in this case the flux of the X-rays is significantly decreased.

The diffracted X-rays were detected using a photon-counting X-ray detector (ASI, Timepix, 512 × 512 pixels) with a pixel size of 55 µm × 55 µm, mounted on the arm of a vertical δ circle. A photon-counting detector with a pixel size of 75 µm (Dectris, Eiger2 X 1M, 1028 × 1062 pixels) was used for the coherent XRD and X-ray diffractive imaging experiments owing to the advantage of the large detection area. In this study, the sample-to-detector distance was approximately 0.75 m, providing an angular resolution of approximately 0.0042° for a photon-counting detector with a pixel size of 55 µm × 55 µm. This is equivalent to the sensitivity of the *d*-spacing estimate of approximately 0.0003 Å. This sensitivity is sufficient to investigate strain relaxation of epitaxial thin films during thermal annealing.

The diffractometer was operated through a six-circle diffraction geometry (Lohmeier & Vlieg, 1993 ▸), and the ϕ and χ angles were adjusted by the motion of the hexapod. However, when the multimodal probe station was mounted on top of the hexapod manipulator, the movement of the ϕ and χ angles was spatially limited. Therefore, ‘2 + 2’ mode is suitable for accessing the *Q* space by combining two detector circles (δ and γ) and two sample circles (θ and μ) (Evans-Lutterodt & Tang, 1995 ▸). Typical powder XRD profiles along the out-of-plane *Q*_*z*_ direction were recorded by scanning vertical δ and θ circles, whereas grazing-incident-angle X-ray scattering (GIXS) profiles were measured by scanning horizontal γ and μ circles. After installing the multimodal X-ray probe station, we tested the diffractometer and available *Q* space.

**Figure 3:**
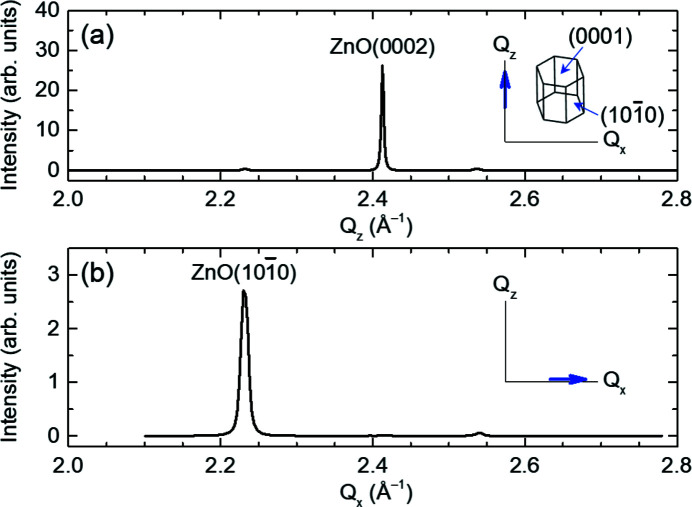
(*a*) Powder XRD and (*b*) GIXS profiles of ZnO nanorods along the out-of-plane *Q*_*z*_ and in-plane *Q*_*x*_ directions, respectively. The GIXS profile is measured at an incident angle of 0.2°. The scan directions in *Q*_*x*_–*Q*_*z*_ reciprocal space are illustrated in the in-set. The (0001) and 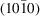 atomic planes of hexagonal wurtzite ZnO are aligned with the *Q*_*z*_ and *Q*_*x*_ directions, respectively.

Representative powder XRD and GIXS profiles are shown in Figs. 3 ▸(*a*) and 3(*b*), respectively. The incident angle for the GIXS measurement was 0.2°. The samples consisted of hydrothermally grown ZnO nanorods (Kim *et al.*, 2015 ▸). The out-of-plane XRD profile shown in Fig. 3 ▸(*a*) exhibits a dominant (0002) Bragg peak denoting the *c*-axis of the hexagonal ZnO. Meanwhile, the GIXS profile in Fig. 3 ▸(*b*) shows a 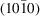 peak corresponding to the *a*-axis of hexagonal ZnO. From the peak positions, the *c*- and *a*-lattice constants of the ZnO nanorods are estimated to be 5.2082 Å and 3.2513 Å, respectively. These values are very close to the bulk values (*c* = 5.213 Å, *a* = 3.253 Å).

**Figure 4:**
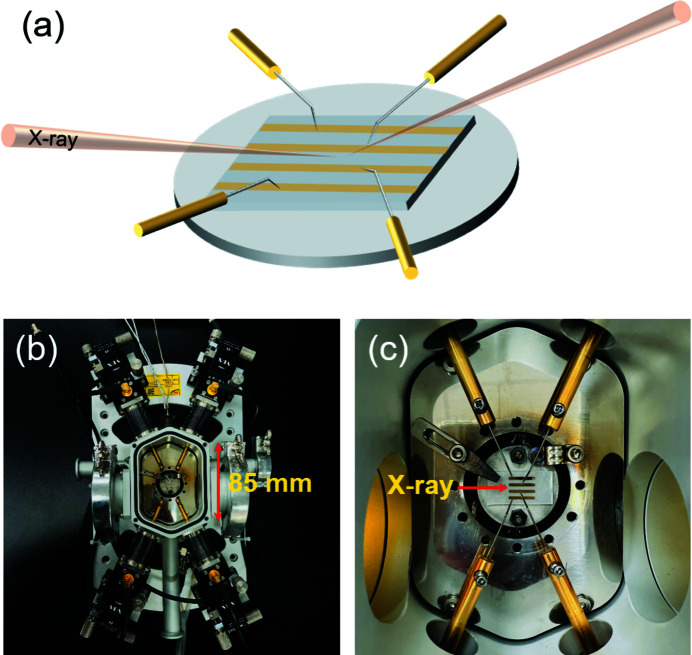
(*a*) Schematic of the operation of four-point probe and XRD measurements. (*b*) X-ray probe station, which weighs approximately 8 kg. Probe holders are adjusted by *x*-*y*-*z* stages. (*c*) Example of sample loaded for multimodal measurements. The probe tips are in contact with metal electrodes.

Fig. 4 ▸(*a*) illustrates how the four-point probe works during the XRD measurements. Figs. 4 ▸(*b*) and 4(*c*) show examples of a four-point probe scheme using a multimodal X-ray probe station. The dimensions of the chamber are shown in Fig. 4 ▸(*b*). A PBN heater with a diameter of 1 inch was located in the center of the chamber and used to control the sample temperatures up to 600°C. The probe tips were in contact with metal electrodes coated on the thin-film sample, where the position of the probe tips could be adjusted using the *x*-*y*-*z* motion stages. A gas control system with a four-channel mass-flow-controller was used to change the gas environment. In addition, coolant lines were built into the chamber walls to minimize outgassing from the chamber during the high-temperature annealing of the sample.

The electrical properties of the films were investigated using a four-point probe method. A two-point probe method was also used. Simultaneous multimodal measurements, which were conducted by adhering to data acquisition protocols, were performed using macro codes in *spec* software. For example, electrical *I*–*V* curves were recorded remotely by controlling the source meter (Keithley 2612B) along with the XRD data in the *spec* files. The beamline can also be used for time-resolved X-ray scattering experiments, which can be demonstrated using the multimodal X-ray probe station.

## 3. *In situ* annealing process of epitaxial ZnO/sapphire(0001) thin films

One of the advantages of the multimodal X-ray probe station is that it can be employed for *in situ* real-time analysis of the annealing process of thin films. For example, XRD profiles and *I*–*V* curves can be measured simultaneously to monitor the structural and electrical resistance changes, respectively (Liu *et al.*, 2018 ▸). This is a simple method to correlate the atomic re­arrangement of a crystal with the activation of electronic transport as a function of annealing temperature (Stuckelberger *et al.*, 2020 ▸; Moon *et al.*, 2012 ▸).

In this study, we analyzed the *in situ* annealing of a 45 Å-thick epitaxial ZnO thin film on a sapphire (0001) substrate. The host sample was deposited using a radio-frequency powder sputtering method as reported previously (Seo & Kang, 2010 ▸). For the four-point probe method, shown in Fig. 4 ▸(*a*), Ti/Au electrodes with thicknesses of 10 and 90 nm, respectively, were deposited on the ZnO/sapphire(0001) thin film using a shadow mask. The electrode was 1 mm wide and 8 mm long. X-rays were illuminated between the second and third electrodes. Time scans were performed while increasing the temperature to monitor the significant change in electrical conductance and the intensity of the ZnO(0002) Bragg peak. The samples were then retained at the desired temperatures for a dwelling time of 1 h to minimize the thermal fluctuations of the ZnO thin films.

**Figure 5:**
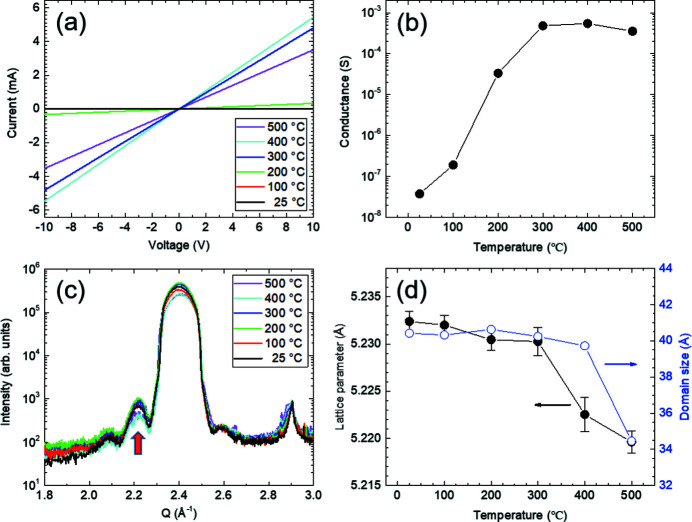
(*a*) *In situ* electrical *I*–*V* curves recorded at 25, 100, 200, 300, 400 and 500°C. (*b*) Variation of electrical conductance as a function of annealing temperature. (*c*) *In situ* microbeam XRD profiles of ZnO thin film at 25, 100, 200, 300, 400 and 500°C. The ZnO(0002) Bragg peak with interference fringes is clear. (*d*) *c*-lattice constant and crystalline domain size of ZnO thin film as a function of annealing temperature. Strain relaxation significantly affects structural and electrical properties after 400°C during annealing.

Fig. 5 ▸(*a*) shows the electrical *I*–*V* curves recorded *in situ* at 25, 100, 200, 300, 400 and 500°C during the heating process. The curves are linear, representing ohmic characteristics. The electrical conductance obtained from the slope of the *I*–*V* curves is summarized as a function of annealing temperature in Fig. 5 ▸(*b*). The electrical conductance increased exponentially up to a temperature of 300°C, and then remained almost constant up to 500°C. Fig. 5 ▸(*c*) shows a series of XRD profiles around the ZnO(0002) Bragg peak during the heating process. Interference fringes owing to finite film thickness were observed. The film thickness was estimated to be approximately 45 Å from the fringe period. The *c*-lattice constant and crystalline domain size of the ZnO thin film were estimated from the peak position and full width at half-maximum of the XRD profiles, respectively. As shown in Fig. 5 ▸(*d*), the peak position and domain size started to decrease significantly after 400°C. The change in *c*-lattice constant after 400°C indicates that strain relaxation cancels thermal expansion. In particular, the interference fringes are weakened after 400°C as indicated by the arrow in Fig. 5 ▸(*c*). The lattice mismatch strain is relaxed by generating dislocations that are strongly correlated with the electrical conductance behavior (Moon *et al.*, 2012 ▸; Mishra *et al.*, 2021 ▸). It is noteworthy that the as-grown ZnO thin film is in a compressive state compared with the bulk *c*-lattice constant of 5.213 Å. This observation was expected because the strained ZnO thin film with a thickness of 45 Å underwent strain relaxation during the annealing process. It can be concluded that the increase in dislocation density owing to strain relaxation causes a constant electrical conductance after 400°C. Our results indicate that the multimodal X-ray probe station is useful for studying the correlation between the structural and electrical properties of semiconductor thin films by performing *in situ* real-time measurements. The applications of this system will be extended to element-specific chemical analyses using X-ray fluorescence and absorption spectroscopy techniques.

## 4. Performance of ZnO nanostructure-based X-ray photodetectors

As X-ray brightness increases in advanced synchrotron facilities, X-ray-induced phenomena such as phase transition, synthesis of nanostructures in aqueous solutions, and photodetectors have been widely studied (Shibuya *et al.*, 2011 ▸; Lee *et al.*, 2019 ▸; Tsai *et al.*, 2020 ▸). The development of X-ray photodetectors using wide-bandgap oxide semiconductors has recently attracted attention because of their outstanding photo-induced electronic transport properties, such as fast responsivity, high detectivity and thermal stability (Zhang *et al.*, 2021 ▸).

**Figure 6:**
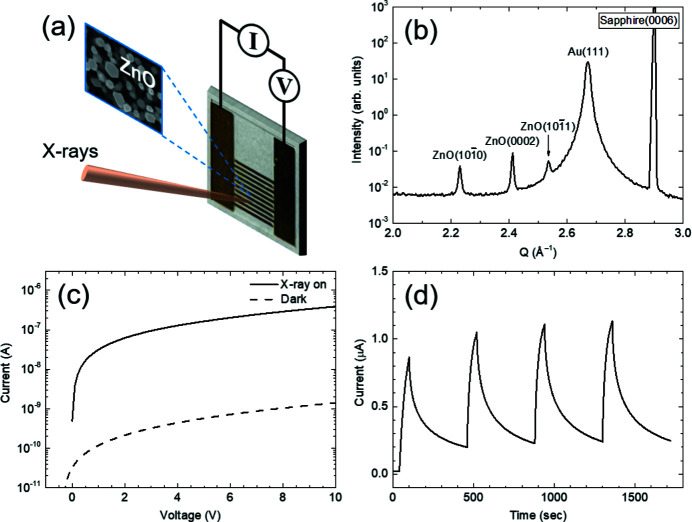
(*a*) Metal–semiconductor–metal type X-ray photodetector. Width and spacing of the metal grids are 200 µm. Top-view SEM image denotes the morphology of the ZnO nanostructures. (*b*) XRD profile of the ZnO nanostructure-based X-ray photodetector. (*c*) X-ray-induced photocurrent and dark current profiles as a function of applied voltage. The photo-to-dark-current ratio at 10 V is approximately 250. (*d*) Sensing profiles for applied voltage of 10 V during repeated X-ray on/off.

In this study, a ZnO nanostructure-based X-ray photodetector was developed, and its performance was investigated using a multimodal X-ray probe station. ZnO nanostructures were deposited on a sapphire(0001) substrate with Ti/Au metal grids using a hydrothermal process to produce a metal–semiconductor–metal type photodetector, as shown in Fig. 6 ▸(*a*). Detailed information on the hydrothermal process of ZnO nanostructures has been given previously (Kim *et al.*, 2015 ▸).

Fig. 6 ▸(*b*) shows a typical XRD profile of the photodetector, confirming the structure of the polycrystalline ZnO nano­structures and Au electrodes. ZnO nanostructures are composed of nanorods with different growth orientations. The top-view scanning electron microscope (SEM) image in Fig. 6 ▸(*a*) illustrates the hexagonal shape of the ZnO nanorods used in this study. Fig. 6 ▸(*c*) shows the X-ray-induced photocurrent and dark current as a function of the applied voltage. A two-point probe method was used to measure the sensing properties. The photo-to-dark-current ratio at 10 V was approximately 250. A photocurrent was generated by exposure to an X-ray microbeam in this study. An open-circuit voltage of −0.35 V at the dark current was observed owing to the large number of charge carriers in the ZnO nano­structures. The sensing characteristics of repeated on/off X-rays are shown in Fig. 6 ▸(*d*). This highlights the sensitive switching of the X-ray-induced photocurrent in the ZnO nanostructures. Raising (τ_1_ and τ_2_) and decay (τ_3_ and τ_4_) time scales were modeled using simple exponential curves. The values of τ_1_, τ_2_, τ_3_ and τ_4_ were approximately 5.48, 29.36, 14.8 and 136.7 s, respectively. These values indicate the slow transport of X-ray-induced photoelectrons across the individual ZnO nanorods. The synthesis and characterization of the ZnO nanostructured X-ray photodetectors will be provided in detail elsewhere (Choi *et al.*, 2022 ▸). Our results demonstrate that the proposed multimodal X-ray probe station is suitable for evaluating the performance of X-ray photodetectors, including their thermal stability, using X-ray microbeams.

## 5. Preliminary results using the Bragg coherent diffractive imaging technique

One of the intended uses of the multimodal X-ray probe station is coherent X-ray scattering measurements including imaging techniques. The 9C beamline aims to support CDI and coherent X-ray micro-beam diffraction experiments; CDI results using a conventional annealing chamber have been recently published (Ahn *et al.*, 2021*a* ▸,*b* ▸; Kang *et al.*, 2021 ▸). Three-dimensional CDI tomography using transmission geometry showed the non-uniform chemical distribution of Pt–Ni nanocrystals and the oxidation state of NiO nanocrystals (Ahn *et al.*, 2021*a* ▸,*b* ▸). Internal deformation of a Cu–zeolite crystal, such as strain development, was investigated using Bragg CDI (BCDI) during the adsorption of hydrocarbon (Kang *et al.*, 2021 ▸). Here, the functionality of the multimodal X-ray probe station in the BCDI technique was tested and the preliminary results are presented.

**Figure 7:**
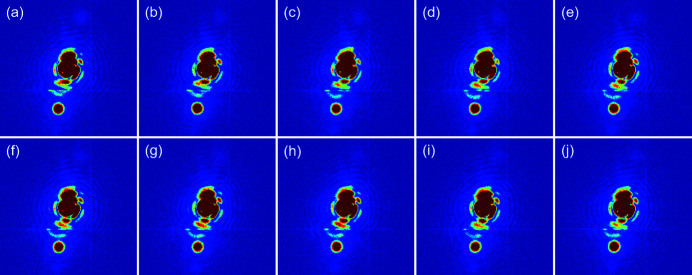
A series of diffraction patterns near the off-specular Au( 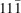 ) Bragg peak. The diffraction patterns of Au nanocrystals were recorded during annealing at 500°C. The interval is Δ*t* ≃ 13 min. Data contain 161  ×  161 pixels corresponding to Δ*Q* = ±0.0237 Å^−1^.

**Figure 8:**
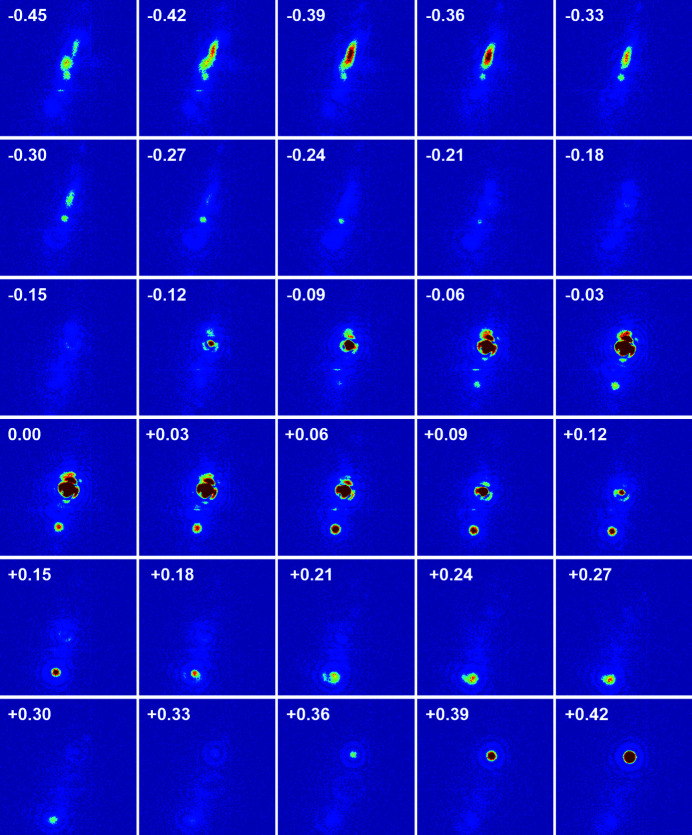
The diffraction patterns of Au nanocrystals were recorded while rotating the sample through Bragg diffraction conditions near the off-specular Au( 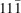 ) Bragg peak. Actually, the θ-rocking angle is ±0.5° with a step of 0.01°. The values shown in the figure represent the rocking angle. Data contain 161 × 161 pixels corresponding to Δ*Q* = ±0.0237 Å^−1^.

Au nanocrystals formed on a sapphire(0001) substrate were prepared by thermal annealing a 20 nm-thick Au thin film through solid-state dewetting. A diffraction pattern around the off-specular Au( 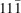 ) peak was recorded. The region of interest in the diffraction patterns is 161 × 161 pixels, corresponding to Δ*Q* = ±0.0237 Å^−1^. The spatial drift was monitored as a function of time at 500°C by recording the time series of the diffraction pattern as shown in Fig. 7 ▸. In addition, the spatial stability of the sample was tested while rotating the sample through Bragg diffraction conditions near the Au( 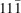 ) peak as shown in Fig. 8 ▸. The θ-rocking angle is ±0.5° with a step of 0.01°. This rocking measurement was repeated for 2 h. A coherent diffraction pattern of Au nanocrystals was clearly observed and the long-term spatial drift was negligible. Based on these results, we conclude that the stability of the multimodal X-ray probe station for coherent diffraction measurements is reasonable. It should be noted that, for the successful performance of BCDI using the multimodal X-ray probe station, there are still many issues to be solved, such as focus size, mechanical stability of the X-ray probe station for ptychography, and photon flux. We believe that this multimodal X-ray probe station will be very useful for performing BCDI on the electric-field-induced strain evolution of patterned thin films.

## 6. Conclusions

In summary, we assembled a multimodal X-ray probe station at the 9C beamline of PLS-II by combining an X-ray diffractometer with a four-point probe station. While observation of structural changes of a sample in response to temperature and gaseous environment is feasible in a general chamber system, one can also observe the response to the applied electric field using the multimodal X-ray probe station. In particular, with the advantage of the multimodal X-ray probe station system, *operando* studies of micrometre-sized electronic devices can be correlated with atomic structures. Understanding electron/ion transport through gate measurements is feasible to conclude the nanometre-scale interfacial stability of metal–oxide–semiconductor-based field effect transistors. This multimodal X-ray probe station was utilized to evaluate the *in situ* annealing of ZnO thin films and the performance of ZnO nanostructure-based X-ray photodetectors. Based on the analyses, we conclude that the multimodal X-ray probe station is suitable for investigating the structural evolution of thin films with simultaneous device performance measurements, particularly for examining the structural phase transitions in highly correlated systems, such as VO_2_ thin films, that involve metal–insulator transitions as a function of temperature. The proposed multimodal X-ray probe station can also be used to investigate the reaction sensitivity of nano-scale two-dimensional materials that correlates with their surface structure. In addition to *in situ operando* capabilities, the probe system can also be utilized for time-resolved X-ray scattering techniques, where the time resolutions are expected to be in the microsecond to sub-nanosecond range.
